# A Genetically Hard-Wired Metabolic Transcriptome in *Plasmodium falciparum* Fails to Mount Protective Responses to Lethal Antifolates

**DOI:** 10.1371/journal.ppat.1000214

**Published:** 2008-11-21

**Authors:** Karthikeyan Ganesan, Napawan Ponmee, Lei Jiang, Joseph W. Fowble, John White, Sumalee Kamchonwongpaisan, Yongyuth Yuthavong, Prapon Wilairat, Pradipsinh K. Rathod

**Affiliations:** 1 Department of Chemistry and Global Health, University of Washington, Seattle, Washington, United States of America; 2 Department of Biochemistry, Faculty of Science, Mahidol University, Bangkok, Thailand; 3 National Center for Genetic Engineering and Biotechnology (BIOTEC), National Science and Technology Development Agency, Klong Luang, Pathumthani, Thailand; Albert Einstein College of Medicine, United States of America

## Abstract

Genome sequences of *Plasmodium falciparum* allow for global analysis of drug responses to antimalarial agents. It was of interest to learn how DNA microarrays may be used to study drug action in malaria parasites. In one large, tightly controlled study involving 123 microarray hybridizations between cDNA from isogenic drug-sensitive and drug-resistant parasites, a lethal antifolate (WR99210) failed to over-produce RNA for the genetically proven principal target, dihydrofolate reductase-thymidylate synthase (DHFR-TS). This transcriptional rigidity carried over to metabolically related RNA encoding folate and pyrimidine biosynthesis, as well as to the rest of the parasite genome. No genes were reproducibly up-regulated by more than 2-fold until 24 h after initial drug exposure, even though clonal viability decreased by 50% within 6 h. We predicted and showed that while the parasites do not mount protective transcriptional responses to antifolates in real time, *P. falciparum* cells transfected with human DHFR gene, and adapted to long-term WR99210 exposure, adjusted the hard-wired transcriptome itself to thrive in the presence of the drug. A system-wide incapacity for changing RNA levels in response to specific metabolic perturbations may contribute to selective vulnerabilities of *Plasmodium falciparum* to lethal antimetabolites. In addition, such regulation affects how DNA microarrays are used to understand the mode of action of antimetabolites.

## Author Summary

Traditional knowledge of gene regulation, learned largely from non-pathogenic model organisms such as *E. coli*, yeast, and mice, suggests that RNA for metabolic pathways are regulated in large part by DNA-binding transcriptional factors that are responsive to cellular metabolic needs. We demonstrate that the malaria-causing *Plasmodium falciparum* parasites, under lethal drug pressure from an antifolate with a known mechanism of action, are incapable of large reproducible changes in RNA levels for the target pathways, or for any other gene throughout the genome. Small RNA changes, possibly informative of perturbed pathways, can be detected in dying parasites. In addition, significant RNA changes are seen when the hard-wired program, governing RNA levels, itself is altered. Our data formally proves that RNA levels for intermediary metabolism in malaria parasites are largely predetermined. We propose that as a parasite with a complex life cycle travels from one largely predictable intracellular biochemical environment to another, such hard-wiring may be sufficient to manage transcript levels for intermediary metabolism without employing sensory functions. Such a system-wide host–parasite difference in gene regulation may create unexpected pharmacological opportunities when important target pathways are rigid in the parasite but dynamically regulated in host cells.

## Introduction

Malaria parasites infect over 300 million people around the world and the most virulent species, *Plasmodium falciparum*, kills 1–2 million individuals per year [1]–[3]. The availability of genome-wide DNA microarrays for *P. falciparum*, has facilitated insights into many complex biological problems. During erythrocytic development, over 3,000 genes are expressed in a cascade of simple, mostly unique, sigmoidal patterns [4]–[7]. While some genes are expressed at a steady rate, hundreds of genes show at least 30-fold change in expression during the erythrocytic cycle [4]. In addition DNA microarrays have been used to understand stage-specific differentiation [8]–[10], to determine invasion preferences [11],[12], and possibly pathogenesis [13]–[16].

At first, it was expected that DNA microarrays would also permit a quick, unbiased look at the mode of action of antimalarial drugs, particularly simple antimetabolites [17]. An underlying premise was that parasites would sense metabolic perturbations from a drug and make compensatory changes in its transcriptome to adjust for the perturbations. Indeed, studies in other organisms have demonstrated the value of such approaches. In *Saccharomyces cerevisiae*, *Mycobacterium tuberculosis*, *Candida albicans*, mammalian cells and even plants, specific antimetabolites up-regulated dozens of target-related RNA by greater than 10-fold [18]–[23]. In many of these systems confidence in the power of DNA microarrays to reveal mechanisms of drug action come from perturbation of well-understood metabolic pathways.

Our early preliminary studies had suggested that RNA levels for metabolic targets in malaria parasites are not sensitive to lethal antifolates nor to resulting specific metabolic perturbations [24]–[27]. Here, in a complete, carefully controlled microarray study, we definitively demonstrate that global gene expression in malaria parasites is regulated in a fundamentally different way from model organisms such as *E. coli* and yeast. Parasite transcription for intermediary metabolism is hard-wired and not responsive to specific, lethal, metabolic perturbations. We further demonstrate that candidate pathways involved in drug-induced death may still be identified through unconventional strategies, including probing for subtle RNA changes with a large number of replicates and tracking alterations in the hard-wired transcription program itself.

## Results

### A defined experimental system

To guard against broad pleotropic transcriptional effects that may be difficult to interpret in drug-treated parasites, our study exploits the potency and specificity of the antifolate WR99210 against *P. faciparum* (Structure, Figure S1). The parasite clone Dd2 fails to proliferate when exposed to 10 nM WR99210 for 48 h [25], [28]–[30]. A concentration of 10 nM was selected because it is enough to kill all sensitive Dd2 cells (EC_50_ = 0.1 nM). Biochemical assays and genetic complementation studies (using human DHFR) have established *P. falciparum* DHFR-TS as the major target of WR99210 [25], [28]–[30].

To identify transcriptional changes that were directly related to death events caused by the lethal effects of WR99210 on DHFR, the present battery of microarray hybridizations included a control WR99210-resistant cell-line, B1G9, which harbors a single integrated copy of human DHFR in a Dd2 background [25],[29]. B1G9 is resistant to as much as 500 nM WR99210.

Finally, to help frame drug-induced changes in RNA levels in the context of cell physiology, Dd2 and B1G9 parasite lines were exposed to 10 nM WR99210 for varying time periods and the effects assessed with respect to clonal cell viability, continued synthesis of nucleic acids, RNA levels for individual genes coding for the effected pathways, and the global transcriptome.

### Robust metabolism and development

**Figure 1:**
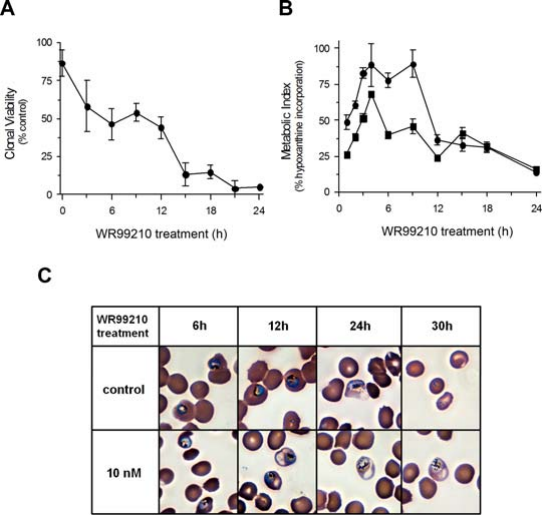
The antifolate WR99210 rapidly triggers commitment to death but fails to shut down metabolism or development. While 10 nM WR99210 is sufficient to commit 50% of the cells to lethality within 6 h, the parasites continue to obey normal metabolic pattern for hypoxanthine uptake and incorporation into DNA (albeit at a lower amplitude) and continue to develop to schizogony for upto 24 h. (A) Rapid decrease in clonal viability of WR99210 exposed Dd2 cells. Trophozoite forms of infected erythrocytes in 10 ml cultures were exposed to 10 nM WR99210 for varying periods. Washed cells were diluted and plated in 96-well plates (see methods). Control diluted cells revealed about 20 positive colonies starting at 12–16 days. (B) Continued incorporation of hypoxanthine in WR99210-treated cells. Young trophozoite forms of infected erythrocytes in 10 ml cultures were exposed to 10 nM WR99210 for varying periods. Cells were pulsed directly with radioactive hypoxanthine for 1 h. Incorporation of radioactive hypoxanthine into DNA was measured by precipitation of nucleic acids on glass fiber filters. Maximum incorporations was seen at 4–8 h into trophozoite development. •; Solvent-treated cells, ▪; WR99210-treated cells. (C) Synchronized Dd2 cells at early trophozoite stage were treated with 10 nM WR99210 or 0.1% DMSO (control) for 48 h. Parasites were visualized by light microscopy of Giemsa-stained blood smears. Images at 6 h, 12 h, 24 h, and 30 h of WR99210 treatment are shown. Based on microscopy, the parasites followed normal development up to about 24 h after WR99210 treatment.

A comparison of biochemical changes, morphological alterations and loss of cell viability in WR99210-treated Dd2 provided the first indication that malaria parasites resisted broad metabolic or developmental arrests in response to specific lethal perturbations. Using clonal viability as a measure of drug-induced death [31], 50% of *P. falciparum* trophozoites became less viable after as little as 6 h of exposure to 10 nM WR99210 (p<0.01) and practically all parasite cells were non-viable after 12 h of drug exposure (Figure 1A). However, even after 24 h of WR99210 treatment, trophozoites continued to follow a preordained metabolic program for converting short pulses of radioactive hypoxanthine into DNA, albeit with a lower amplitude (Figure 1B). Microscopic examination of WR99210-treated trophozoite forms of the parasite failed to show morphological changes until about 24 h after treatment when the schizonts appeared unhealthy (Figure 1C). At subsequent hours, control cells released merozoites and generated healthy rings but the WR99210-treated parasites remained as ill schizonts. In parallel assays, WR99210-resistant B1G9 cells behaved like untreated Dd2 (data not shown).

### Tracking genome-wide changes

**Figure 2:**
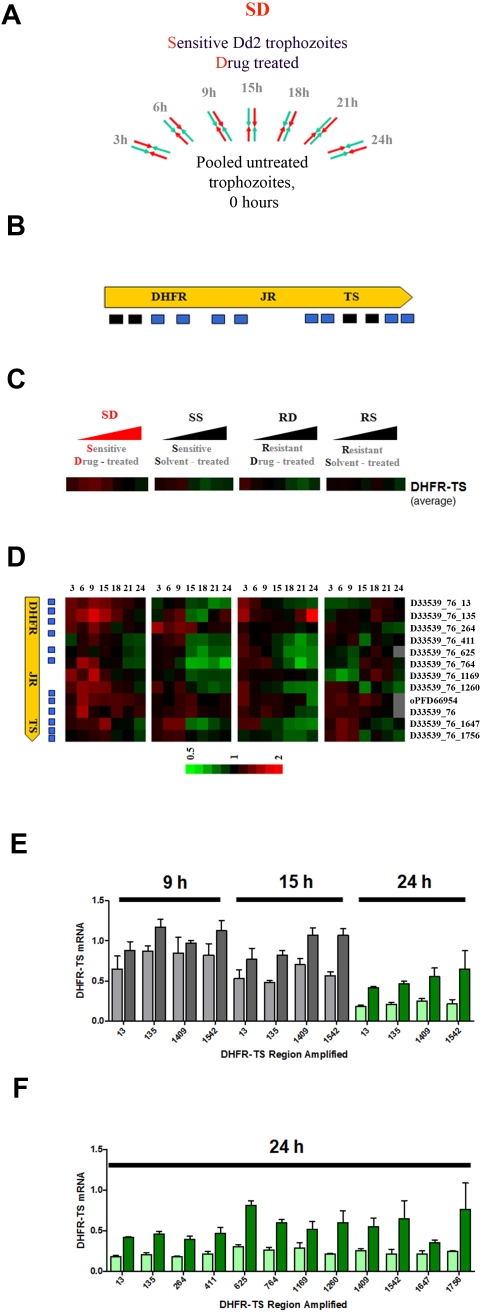
RNA for the antifolate target dihydrofolate reductase-thymidylate synthase is not overexpressed in WR99210-treated cells. Using multiple probes for DNA microarrays, as well as multiple primer sets to amplify different regions of the DHFR-TS cDNA by qRT-PCR, it is shown that mRNA for DHFR-TS is not overexpressed in protective amounts after antifolate treatment. Note that the microarray data color bar has been set at high sensitivity to pick up any small but significant and reproducible changes in expression. (A) Design of the DNA microarray experiment. This simple scheme was repeated twice for each combination of treatments (WR99210 or DMSO solvent) and varying parasite clones (Dd2 vs B1G9) in Seattle. Young trophozoites were split into treated cells (3 h to 24 h), and untreated cells (0 h). After WR99210 treatment at 10 nM concentrations for various durations, RNA was extracted, converted to cDNA, fluorescently labeled, and hybridized against RNA from pooled reference untreated cells (0 h). One, duplicates from the same biological samples were run with dye inversion. Two, the exact scheme was repeated with independently grown parasites, thus generating 4 hybridizations per time point of WR99210 treatment. This scheme was labeled *SD*, *for Sensitive* cells treated with the *drug* analog WR99210. Finally, the whole scheme of duplicates, was repeated 3 more times (*Sensitive* clone Dd2 with *Solvent* (SS), *Resistant* clone B1G9 with the *drug* analog WR99210 (RD), and *Resistant* clone B1G9 with *Solvent* (RS). Altogether this resulted in microarray data generated from over 123 slides, each carrying about 7,685 oligonucleotide probes. Details of the large experiment are presented in the Methods section. (B) Illustration of regions of DHFR-TS gene queried with oligonucleotide probes (black plus blue boxes) and qRT-PCR primers (black boxes) to measure DHFR-TS mRNA levels in parasites treated with the antifolate WR99210. The DNA sequences corresponding to the 12 unique microarray oligonucleotide probes for DHFR-TS are presented in Table S1. The DNA sequences for the primers used for DHFR-TS qRT-PCR are presented in Table S2. (C) WR99210-treated sensitive cells do not increase DHFR-TS RNA to high levels, as judged by DNA microarrays data that was collected and averaged from 12 different, unique DNA microarray probes representing different parts of the complete DHFR-TS coding region (see Figure 3B). Hybridization of fluorescent cDNA samples originating from parasites treated with WR99210 for various times (red fluorescence) were compared to non-treated parasites (green fluorescence) and represented as R/G ratios on a log2 scale: A black box signifies no change in expression compared to starting trophozoites, a red box represents some over-expression and a green box designates a decrease in DHFR-TS RNA, compared to starting trophozoites (time 0 h). Missing data is shown as gray. Each bar (SD, SS, RD, and RS) represents different combinations of cell clones (S or R) and treatment (D or S), starting from 3 h to 24 h of treatment. (D) Individual DHFR-TS probes revealed no significant differences in RNA levels from within the DHFR-TS coding region. (E) WR99210-treated sensitive cells do not increase DHFR-TS RNA to high levels as judged by qRT-PCR (3–24 h treatment). To independently measure the magnitude of DHFR-TS RNA levels in antifolate treated Dd2 cells, qRT-PCR was applied to 4 different regions of the DHFR-TS coding mRNA sequence. The primer pairs from around nucleotide 13, 135, 1409, and 1542 of the coding region, confirmed that during the 24 h maturation of DHFR-TS, solvent-treated parasites showed a gradual decrease in DHFR-TS RNA. Furthermore, while the qRT-PCR revealed a slightly higher level of DHFR-TS RNA in WR99210-treated Dd2 cells at 24 h, this was not due to an increase over the RNA present before initiation of drug treatment. DHFR-TS mRNA level are represented for solvent-treated cells at 9 h and 15 h (gray bar), for solvent-treated cells at 24 h (light-green bar), for WR99210-treated cells at 9 h and 15 h (black bar), and for WR99210-treated cells at 24 h (dark-green bar). Data are shown as means±SEM. (F) DHFR-TS RNA, probed across the whole coding region, failed to show up-regulation in WR99210-treated Dd2 cells at 24 h. There was a very small but consistent and significant failure to undergo normal repression at 24 h. However, the failure to overexpress protective quantities of DHFR-TS was consistent with the DNA microarray data. DHFR-TS mRNA level in solvent-treated cells (light-green bar) and in WR99210-treated cells (dark-green bar) are shown as means±SEM.

In a single large controlled experiment, *Plasmodium* transcriptome changes were followed in WR99210-treated parasites using DNA microarrays with 7,685 oligonucleotide probes per slide, representing all open reading frames in the genome [4],[17]. For added value and confidence, the custom array also carried *multiple probes per gene* for key enzymes in the target pathway of folate and pyrimidine metabolism (Table S1 and S3). RNA samples from synchronized Dd2 and B1G9 trophozoites, that had been treated with 10 nM WR99210 for varying durations, were hybridized against a common pool of trophozoite RNA from a cognate clone (Figure 2A). For each time point, samples from biological duplicates were hybridized to four microarray slides, including dye exchanges. The biological duplicates were from independently propagated cultures to minimize misleading, stochastic variations in gene expression for surface proteins ([32], Figure S2 and Figure S3 for description of data normalization and variation between technical and biological variability). In total, the present normalized data is derived from 123 microarray hybridizations. As discussed below, this redundancy and accuracy was necessary to interpret some small but informative perturbations in RNA in *P. falciparum*. For some genes, it was possible to detect as little as 10–20% changes in RNA levels, with statistically significant reproducibility. Details of the experimental design, raw output, and statistical analysis are presented in MIAME-compliant format to the NIH-based GEO database (Accession # GSE9724 for WR99210 data from Seattle and # GSE9853 and # GSE9868 for the pyrimethamine data from Bangkok).

### “Unresponsive” target pathways

During normal 48-hour developmental changes in erythrocytes, RNA levels for individual enzymes for pyrimidine and folate biosynthesis change nearly 10-fold [4]. During continual WR99210 treatment for 24 h, malaria parasites showed very little deviation in their transcriptome, even as they died from antifolate treatment.

First, expression of DHFR-TS, the immediate target of antifolates was examined in detail. Fluorescently labeled cDNA, generated from WR99210-treated and non-treated cells, was hybridized to 12 unique oligonucleotides derived from different parts of the 1,863 bp DHFR-TS coding strand (Figure 2A and 2B). Regardless of the WR99210-susceptibility status of the parasite clone, and regardless of whether the cells were treated with solvent or the antifolate, hybridization of fluorescent cDNA to most probes for DHFR-TS did not increase (and actually decreased slightly) during the 24 h normal progression of trophozoites to schizonts, (Figure 2D). Based on combined data from all 12 probes, RNA coding for DHFR-TS did not increase by more than 20% at any time point after WR99210-treatment (Figure 2C). This microarray-based analysis of DHFR-TS is consistent with earlier limited measurements using RNA Protection Assays (RPA) [25] and qRT-PCR [33]. For additional certainty, given recent description of differential expression of small regulatory RNA within the coding region of mammalian DHFR [34],[35], we undertook an independent detailed analysis of DHFR-TS expression using qRT-PCR spanning twelve different parts of the DHFR-TS coding region. As seen with the different unique oligonucleotide microarray probes (Figure 2D), qRT-PCR (Figure 2E–F) unambiguously confirmed that DHFR-TS RNA, or parts of DHFR-TS RNA, were not overproduced at protective levels in parasites treated with the lethal antimetabolite WR99210. Since the 10 nM WR99210 treatment was at 100-times the EC_50_ for Dd2 (0.1 nM), complete protection through increases in target RNA levels would require at least 100-fold increases in DHFR-TS RNA. Even a 10% protection from a RNA-based mechanism would require at least a 10-fold increase in DHFR-TS RNA.

**Figure 3:**
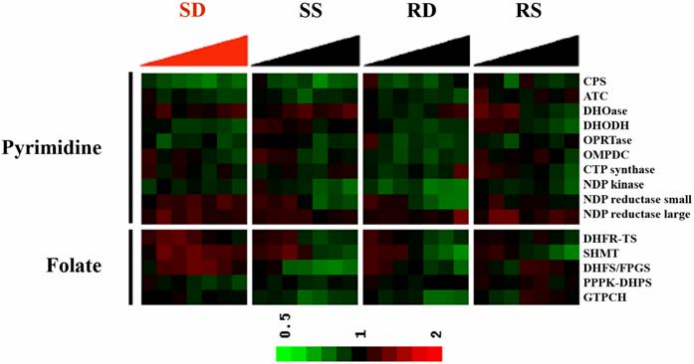
As seen for DHFR-TS RNA levels, RNA for other enzymes in the folate and pyrimidine biosynthesis do not increase to high levels in WR99210-treated sensitive-parasites, Dd2. DNA microarray data was examined, not just from the coding region of the known antifolate target DHFR-TS (Figure 2), but also from the coding sequences for all the known enzymes of de novo pyrimidine and folate metabolism. Each gene was probed with at least two unique oligonucleotide probes (see Table S3 for oligonucleotide sequences). As in Figure 2C, hybridization of fluorescent cDNA originating from parasites treated with WR99210 for various times (red fluorescence) was compared to non-treated parasites (green fluorescence) and represented as R/G ratios on a log2 scale: A black box signifies no change in expression compared to starting trophozoites, a red box represents some over expression and a green box designates a decrease in DHFR-TS RNA, compared to starting trophozoites (time 0 h). Each bar (SD, SS, RD, and RS) represents different combinations of cell clones (S or R) and treatment (D or S), starting from 3 h to 24 h of treatment.

Second, in addition to DHFR-TS, the expression of fourteen additional enzymes in the folate and pyrimidine biosynthesis pathways also did not deviate significantly from their normal transcriptional patterns. This analysis was based on at least two different microarray probes for each of the enzymes listed in Figure 3. The only two RNA species that showed small, consistent, significant changes from their normal expression program were serine hydroxymethyltransferase (SHMT) and a ribonucleotide reductase small subunit gene (RNR2). However, even these two responsive RNA species showed, at most, only a 2-fold change at very late time points (maximum 105% increase at 24 h, p<0.002). Even though the methylenetetrahydrofolate-using thymidylate synthase is thought to be the ultimate target of DHFR inhibition, the unresponsiveness of pyrimidine biosynthesis to DHFR-TS inhibitor have not previously been reported. Just as our preliminary microarray data had reported [24],[26],[27], a qRT-PCR study from an independent group has also shown that RNA for folate-biosynthesis enzymes are not up-regulated in response to pyrimethamine-treatment [33]. This other study did not look at changes in RNR2 and did not pickup the subtle drug-dependent alterations in transcripts for SHMT that are seen with the current large microarray data set.

### “Unresponsive” genome

Given the inability of *P. falciparum* transcriptional program to overproduce RNA for DHFR-TS and related enzymes after antifolate treatment, we wanted to understand the extent of the overall transcriptional obstinacy in drug-sensitive parasites. In the first 3 h after WR99210 treatment, when death events were underway (p<0.05, Figure 1A), no genes showed statistically significant deviations from their normal developmental program that was greater than 2-fold in expression. Even 24 h after WR99210-treatment, when all cells are completely committed to die, greater than 99% of the genes in the *Plasmodium* transcriptome did not deviate significantly in gene expression.

### Death-related “RNA whispers”

By exploiting our experimental design, which included side-by-side treatment of isogenic sensitive and resistant malaria parasite cell lines with a potent, specific, and lethal antifolate, it was possible to detect small reproducible changes in RNA in dying cells and only in dying cells.

**Figure 4:**
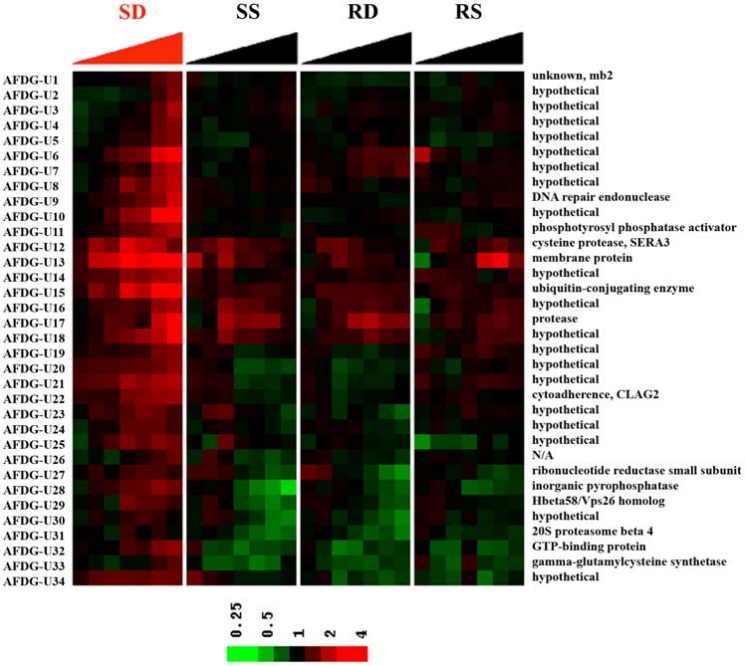
Death-related up-regulated expression changes in WR99210-treated parasites. Genes whose expression changed in dying cells but not in three other controls were identified. Expression levels were compared between SD and SS, SD and RD, and SD and RS at each time point. Probes were selected against these three comparisons jointly, with multiple testing adjusted *p*<0.01. Thirty four genes showed a drug-dependent increase in gene expression (Table S4 for PlasmoDB gene number).

**Figure 5:**
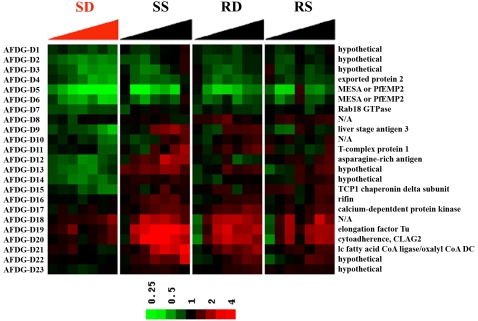
Death-related down-regulated expression changes in WR99210-treated parasites. Genes whose expression changed in dying cells but not in three other controls were identified. Expression levels were compared between SD and SS, SD and RD, and SD and RS at each time point. Probes were selected against these three comparisons jointly, with multiple testing adjusted *p*<0.01. Twenty one genes showed a drug-dependent decrease in gene expression (see Table S5 for PlasmoDB gene number).

Out of 7,685 oligonucleotides examined, there were 34 genes whose expression levels increased at least at one time point during the 24 h WR99210 study (Figure 4, and Table S4 for gene names, gene ID numbers, and fold-changes). There were 21 genes whose expression levels decreased at least at one time point (Figure 5 and Table S5). These changes were considered antifolate death-related genes (AFDG) because they were not seen in solvent-treated, drug-sensitive Dd2 parasites, and were not seen in solvent-treated or WR99210-treated resistant B1G9 cells.

The numerous death-related RNA changes were subtle and probably not protective, but they were statistically significant and possibly reflected larger physiologically important metabolic perturbations in dying parasite cells. The death-related transcripts provide the first insights into downstream events that may connect inhibition of DHFR-TS by antifolates to ultimate loss of cell viability (Table S4 and S5 for PlasmoDB gene numbers). Given the known mechanism of action of antifolates, the presence of ribonuceotide reductase and DNA repair endonuclease on this list was satisfying. The intriguing presence of putative cell-signaling proteins (phosphotyrosine phosphatase activator; a GTP binding protein; a Rab18 GTPase; and a calcium-dependent protein kinase) and some potential degradative enzymes (cysteine protease SERA3; protease; 20S proteosome beta 4) on the list of death-related genes should stimulate investigations of their possible role in antifolate toxicity. The list of death-related genes also included some known enzymes whose role in antifolate-mediated death is not obvious but should also be of interest (inorganic pyrophosphatase; gamma-glutamylcysteine synthetase; elongation factor Tu; and long-chain fatty acid ligase-oxalyl CoA decarboxylase). Finally, this genome-wide analysis revealed that, of the 55 antifolate-triggered small transcript changes, there were 25 hypothetical gene products with no previous known functions in other cell types.

### Confirmation and cross-checks

To evaluate the validity and reliability of the small changes in RNA levels detected in the DNA microarray experiments, two different approaches were taken.

Conventional quantitative RT-PCR was used to compare RNA expression in independently cultivated *P. falciparum* Dd2 cells, before and after 10 nM WR99210 treatment for 24 h. This time point was used because the most significant changes on the microarray occurred after 24 h and the magnitude of the changes approached the resolution limits of RT-PCR. Out of 3 randomly chosen up-regulated genes, all 3 showed the expected small up-regulation upon WR99210 treatment (Table 1). Out of 3 randomly chosen down-regulated genes, 2 showed down-regulation as expected but one did not. Overall, we concluded that, though small, most RNA “whispers” picked up by the DNA microarrays were verifiable by qRT-PCR.

**Table 1:** qRT-PCR cross-check of representative microarray data.

Present label	PlasmoDB ID	Description	Microarrays			qRT-PCR		
			Ratio relative to time 0		SD/SS	Ratio relative to time 0		SD/SS
			SD24	SS24		SD24	SS24	
AFDG-U13	PFD1120c	membrane protein	3.42	1.03	3.31	2.82	0.98	2.89*
AFDG-U15	PF10_0330	ubiquitin-conjugating enzyme	2.70	1.14	2.37	3.18	0.82	3.89
AFDG-U28	PFC0710w	inorganic pyrophosphatase	1.51	0.44	3.42	1.65	0.29	5.75
AFDG-D9	PFB0915w	liver stage antigen 3	0.42	1.50	0.28	1.23	3.25	0.38
AFDG-D11	PFB0635w	T-complex protein 1	0.68	2.01	0.34	1.70	1.46	1.17*
AFDG-D21	MAL6P1.231	lc fatty â CoA ligase/oxalyl CoA DC	0.90	3.77	0.24	0.86	1.95	0.44*

*p-value* of microarray data and half of qRT-PCR were <0.05. * *p*<0.16.

**Figure 6:**
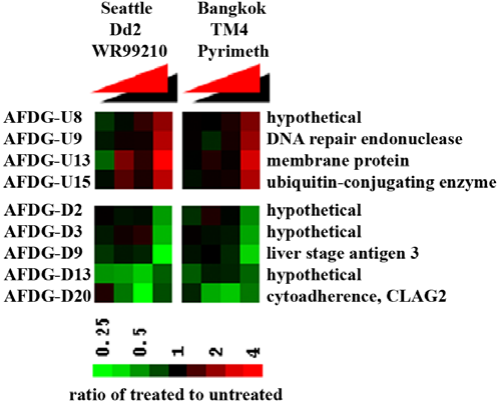
Independent DNA microarray-based confirmation of death-related expression changes in antifolate-treated parasites from two different labs. Early trophozoites (approximately 20 h post invasion) were treated with antifolates (10 nM WR99210 for Dd2 and 0.5 µM pyrimethamine for TM4/8.2) for varying periods. RNA was isolated and converted into labeled cDNA, and hybridized to a 70-mer microarray as described in the Methods section. The expression ratio of antifolate-treated to untreated control (SD/SS) at each time point was plotted. WR99210 data at 3 h, 6 h, 9 h, and 24 h were compared to pyrimethamine data at 2 h, 4 h, 8 h and 24 h.

In a completely different approach, two partner labs from two different parts of the world compared microarray data to determine if antifolate-treated parasites shared some common signatures in their transcriptome. Realistically, all the WR99210-reponsive genes from the Seattle study were not expected to show up in the Thailand study because the two groups were working with independently printed arrays, different parasites strains (Dd2 vs TM4/8.2), different antifolates (WR99210 vs pyrimethamine), and different treatment antifolate doses (99.9% vs 50% IC values). Yet, some of the most relevant changes should be shared, given the common mechanism of action of WR99210 and pyrimethamine. Indeed, a genome-wide comparison of transcript differences in antifolate-treated parasites versus solvent-treated parasites not only revealed 4 genes that were up-regulated and 5 genes that were down-regulated in concordance but the changes occurred at approximately the same time after antifolate treatment (Figure 6). In addition to some new proteins with no prior known functions, this set included a DNA repair endonuclease (MAL13P1.346) and a ubiquitin-conjugating enzyme (PF10_0330). It is not clear why the drug-induced down-regulation of RNA for the cytoadherance linked protein CLAG2 (PFB0935w) [36],[37] was seen in this set, but it appeared consistently in all other measures of antifolate toxicity in the present microarray study.

### Altered hard-wiring

**Figure 7:**
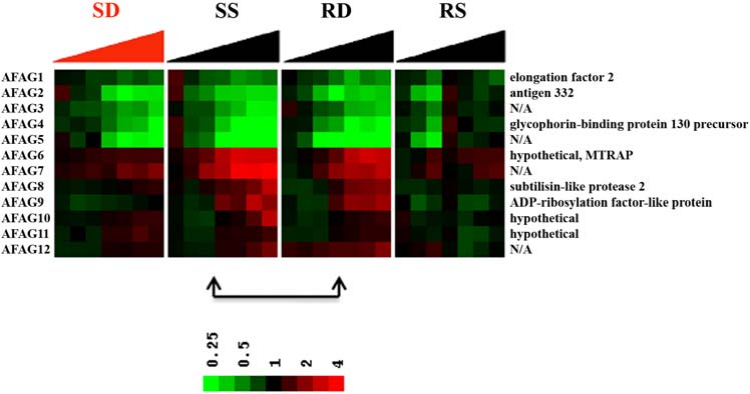
Adaptive changes in *P. falciparum* under long-term WR99210 exposure. Genes in resistant clone B1G9 under drug pressure (RD) acted like sensitive clone Dd2 without drug pressure (SS). Expression levels of RS were compared with SS and RD (RS vs SS and RS vs RD) at corresponding time points and correlation coefficient (*r*) were computed between SS and RD. Probes that met the criteria of *p*<0.01 for both comparisons and *r*>0.85 were selected (Table S6 for PlasmoDB gene number).

It was hypothesized that even if a genetically determined hard-wired transcriptome is insensitive to real-time arrival of antimetabolites in the cell, perhaps the hard-wired program itself may evolve to tolerate an antimetabolite, particularly if given a chance to adapt over successive generations. Indeed, in the present large experiment with multiple controls, it was possible to identify genes whose expression in the resistant B1G9 cells was rewired in concordance with protection against the drug WR99210 (Figure 7 and Figure S4).

In this most compelling gene set, expression in the drug-resistant B1G9 cells behaved normally (as in non-treated, drug-sensitive Dd2 cells) *only when B1G9 cells were under WR99210 pressure* (Figure 7 and Table S6). In other words, expression of these genes was rewired so that it matched normal cells only when the antifolate WR99210 was present. The expression pattern of these genes was dissimilar in dying, drug-sensitive Dd2 cells exposed to WR99210, or resistant B1G9 cells grown without WR99210 (Figure 7). This data set included up-regulation of subtilisin-like protease 2 (PF11_0381) which might promote egress and reinvasion [38]–[42], an ADP-ribosylation factor-like protein (PFI1005w) [43],[44], and several “hypothetical proteins”. While detailed follow up studies will clearly be needed, the alteration in subtilisin-like protease expression ties nicely with morphological arrest of WR99210-treated parasites as ill schizonts and failure to see reinvasion and rings at later time points (Figure 1C).

A second set of permanent genetic alterations in the transcription program involved genes whose expression in the resistant B1G9 cells was different from the WR99210 sensitive Dd2, regardless of whether the antifolate was present or not (Figure S4 and Table S7). While it is possible that these genes also contribute to antifolate resistance mechanisms, this second data list should be accepted with caution since at least some of the RNA changes may have arisen through adventitious changes in the cell line due to collateral genetic damage during WR99210 adaptation.

## Discussion

Many commonly accepted paradigms for designing selective antimetabolites originate from the study of antifolates. Prior to the availability of genome sequences, mechanisms of drug action and the role of active site in selectivity were identified through intuitive comparisons to normal metabolites [45] and validation through resistance and transfection [46]–[48]. Our understanding of selective and potent antimetabolites continues to improve: Low, fixed levels of target enzyme in the parasite and the selective ability of host cells to overexpress target enzymes can play an important role in drug selectivity [25]. While useful, all such approaches have always relied on existing knowledge of biochemistry, metabolism, and pharmacology. Now, with the availability of genome sequences for *Plasmodium* parasites [49]–[51], and accompanying tools such as DNA microarrays [52],[53], there is much enthusiasm about using open ended tools to decipher drug action [17], particularly downstream biochemical and cellular events that lead to cell death.

First, the present study shows that WR99210 treatment of *P. falciparum* does not trigger overexpression of RNA for DHFR-TS, the known target of the antifolate (Figure 2). Casual observation of the microarray data clusters may suggest that DHFR-TS levels increase slightly. However, the color representations can be deceiving, in part because they are set at very high sensitivity (Figure 2D). The actual DHFR-TS increases are neither large nor statistically significant. While the follow up quantitative RT-PCR (Figure 2E and 2F) did show slightly higher levels of DHFR-TS RNA in treated cells, this was not due to RNA induction. The small “increases” in DHFR-TS RNA level in WR99210-treated Dd2 cells arise from a slight delays in normal degradation of DHFR-TS RNA, mostly at very late time points in the dying cell. When one asks the big question, do malaria parasites overexpress the target DHFR-TS RNA to protective levels when treated with the antifolate WR99210? The answer is clearly negative. Can DNA microarrays be used to unambiguously assign mechanisms of antifolate action through real-time changes in RNA levels after drug exposure? The answer, again, is unambiguously negative.

Beyond the immediate target DHFR-TS, the parasites also do not overproduce RNA for any of the enzymes of pyrimidine or folate metabolism, two pathways known to be effected by antifolates (Figure 3). Most importantly, long after *P. falciparum* cells treated with WR99210 were committed to death, there were no large consistent reliable increases in RNA for any of the genes in the *P. falciparum* genome. The last observation shows that the transcriptional obstinacy of *P. falciparum* is not just restricted to folate biochemistry but permeates through much of the parasite's metabolic network involved in control of cell proliferation, and eventually cell death. Since this large project started and has been reported in preliminary form at scientific meetings, several other smaller DNA microarray studies have also encountered transcriptomes resistance to antimetabolites. This includes work on the antimalarial choline analogue T4, the mitochondrial inhibitor atovaquone, and most recently the polyamine biosynthesis inhibitor DFMO [54]–[56]. Earlier claims that malaria parasites show specific large transcriptional responses to chloroquine [57], were reversed [58]. The later conclusion appears to be correct since an independent study has also recently claimed a lack of real-time changes in RNA levels in chloroquine-treated *P. falciparum* [59]. Doxycycline a protein synthesis inhibitor for subcellular organelles caused a whole-sale shutdown of apicoplast RNA, not specific up-regulation of RNA for the target protein [60]. The emerging general consensus is that malaria parasites do not mount large increases in RNA in response to antimetabolites. Our conclusions from the original discovery using antifolates remains compelling because the study uses a potent, specific inhibitor (WR99210) that targets a genetically validated target (DHFR): Drugs with broad or poorly defined mechanism of action were avoided because they can add to existing uncertainty about malarial transcriptional responses.

Even though large protective changes in RNA were not seen in drug-treated malaria parasites, the tightly controlled nature of the present study led to unbiased glimpses into small, subtle downstream RNA changes in drug treated malaria parasites. One type of change involved small, reproducible real-time changes in RNA in sensitive cells and only in the sensitive cells (Figure 3, 4 and 5). These include RNA coding for proteins involved in relevant target pathways: ribonucleotide reductase of nucleotide metabolism and serine hydroxymethyltransferase of folate metabolism. In addition, there were dozens of new genes whose expression was perturbed and whose role in folate pharmacology would be new and unexpected, including enzymes involved in DNA replication, cell signaling, and protein turnover. Secondly, while the hard-wired transcription for metabolic genes in malaria parasites was largely unresponsive to drug-treatment in real-time, the hard-wiring program itself could evolve in a population that is under continual drug pressure. Such alterations in the transcriptomes offer glimpses into new biochemistry related to drug action (Figure 7). The last finding presents a clear caution to malaria scientists who rely heavily on DNA transfections to study drug action. Transfection of the malarial parasite line Dd2 with single copy of the human DHFR originally proved the primary mechanism of action of WR99210 [29]. However, emergence of transformants always involve long delay phases, very similar to those seen during in vitro selection for drug resistance in the laboratory [61],[62]. It is very likely that while human DHFR helps confer resistance to WR99210, additional genetic changes in the hard-wiring of gene expression are necessary to fully realize the WR99210 tolerance and optimum growth in the transformed cell. Identification of these secondary genetic changes are expected to be fertile grounds for fully understanding downstream biochemical changes that lead from DHFR-TS inhibition to cell toxicity.

The present findings add some general rules to help us understand how some good antimalarial drugs work. First, as gene amplification can contribute to drug resistance [63], unconditional suppression of target protein and RNA for metabolic enzymes may contribute to unusual vulnerabilities in the parasite. Previously, *P. falciparum* DHFR-TS protein was shown to bind its cognate RNA sequence differently than the host protein [25]. Now we show that the production of RNA itself may be severely limited and this repression of transcription is largely insensitive to metabolic changes. Second, in principle, while natural metabolites and their toxic homologues may not induce large scale changes in target gene expression in malaria parasites, it is conceivable that some other antimalarial molecules could exhibit broad toxicity, in part, by misdirecting the hard-wired transcriptional program of *P. falciparum*. Third, if there is limited flexibility in regulating gene-expression, perturbations by drugs must be balanced by compensating mutations affecting the transcriptome [64]. This would influence frequencies of drug resistance in unpredictable ways [62],[65], as well as leave molecular footprints of prior drug exposure throughout the genome [66],[67].

The evolutionary implications of the hard-wired malaria parasite transcriptome to control metabolism are significant. Parasites appear to have at least two fundamentally different strategies for gene regulation: Alterations in expression of genes for surface proteins occur randomly to help parasites “outmaneuver” unpredictable immune responses from each new host the parasite encounters [68]–[70]. In contrast, for intermediary metabolism, the obligatory parasites seem to have evolved a deterministic transcriptional program to match the defined and predictable biochemical makeup of host cells. Compared to variations in immune responses, the biochemical environment between different host individuals probably does not vary significantly. The biochemical adaptations which accompany these evolutionary choices in gene regulation must be significant. *A priori*, the systematic, sequential, rhythmic expression of metabolic genes in malaria parasites must be determined by a sequential expression of regulatory molecules which are largely insensitive to intracellular levels of important metabolites. Of course, while such a model could apply to repeated erythrocytic cycles, it need not preclude a signal-based strategy for influencing differentiation of parasites.

## Materials and Methods

### DNA microarrays for *P. falciparum*

The present experiments used spotted DNA microarrays [71] that were fabricated as previously described [9],[32]. Commercially available malaria oligonucleotides (Operon, version 1.1, https://www.operon.com/arrays/oligosets_malaria.php) were combined with in-house oligonucleotides representing *P. falciparum* genes in folate and nucleic acid metabolism. The in-house oligos were designed at the University of Washington using ArrayOligoSelector [52] and synthesized by Operon Biotechnologies, Inc. The arrays, each representing the majority of malarial open reading frames plus the custom oligonucleotides, were printed on polylysine-coated slides using an ultra fast, linear servo driven DeRisi microarrayer. Slides were post-processed and hybridized following the protocols as previously described (http://cmgm.stanford.edu/pbrown/protocols/index.html). Each platform used in the following experiments is described and can be accessed at the NIH-based GEO database (Accession # GPL6187 for WR99210 data from Seattle and # GPL6187 and # GPL6269 for the pyrimethamine data from Bangkok).

### Parasites

At the University of Washington, Seattle, WA, *P. falciparum* clones Dd2 and B1G9 were used for treatment with WR99210. Clone Dd2 was derived from clone W2 from Southeast Asia and is resistant to a variety of antimalarials, but not WR99210 [25],[65],[72]. The isogenic clone B1G9, which harbored a single integrated copy of human DHFR in a Dd2 background, conferred resistance to WR99210 and was kindly provided by Drs. David Fidock and Thomas Wellems [25],[29]. At the National Center for Genetic Engineering and Biotechnology (BIOTEC), Bangkok, Thailand, a pyrimethamine-sensitive laboratory clone TM4/8.2 was treated with pyrimethamine prior to microarray analysis. The TM4/8.2 clone was obtained from Dr Sodsri Thaithong, Chulalongkorn University, Thailand. At both sites, parasites were cultured *in vitro* by standard methods [73].

### Clonal viability

Clonal viability of WR99210-treated parasites was based on a previously established assay [31]. Forty 10 ml cultures, with synchronized early-trophozoite forms of infected erythrocytes, were treated for different durations with 10 nM WR99210 in a final concentration of 0.1% DMSO. Treatment times ranged from 3 h to 24 h, with two flasks per time-point. At appropriate times, cells from each flask were washed and clonally diluted in drug-free medium. Representative dilutions from each flask were plated at a density of about one infected erythrocyte per well in 24 wells of a 96-well plate (4 samples per time point). Growth in each well was monitored microscopically in drug-free medium for two to four weeks. On average, 20 “colonies” (i.e. parasite containing wells) were identified from solvent treated, time-zero parasite populations.

### Rates of DNA metabolism

Trophozoite forms of infected erythrocytes in ten ml cultures were incubated with 10 nM WR99210 for 1–24 h and then pulsed with radioactive hypoxanthine (2 µCi per flask, 22 mCi/umol) for one hour prior to collection of precipitable radioactive DNA on glass fiber filters. In the absence of drug treatment, maximum incorporation was seen at 4–10 h into trophozoite development (approximately 5,000 cpm; 500 pmol/flask).

### Drug treatment and RNA extraction

Overall, in Seattle, for the master DNA microarray experiment, two large populations of Dd2 cells and two large populations of B1G9 cells were prepared for treatment with WR99210. Dd2 clones, starting with about 100 infected erythrocytes per flask, were setup in 60 flasks and propagated until 30 ml cultures were established at 5% parasitemia, 2% hematocrit in each flask. These independently propagated parasites were pooled and redistributed into 60 flasks before drug treatment (see below). A second set of biological replicates of Dd2 were propagated, pooled, and split independently to give two truly independent populations of Dd2. The overall goal was to neutralize stochastic changes in gene expression for surface proteins [32]. This whole exercise was repeated separately with clone B1G9.

To describe the details of the parasite preparation, in each set above (each biological replicate of Dd2 or B1G9), parasites were initially seeded in 10 ml cultures with about 100 infected erythrocytes per flask. When parasitemia reached about 5%, the cells were transferred to 30 ml cultures. When parasitemia reached 5% in each 30 ml culture the first time, the infected cells were synchronized with 5% sorbitol [74]. When parasitemia reached 5% again, and when most of the infected erythrocytes were in the ring stage, the synchronization was repeated once more. These parasites (in one set) were pooled and redistributed into 30 ml flasks, until they reached early trophozoite stage. Half of these samples were saved as “Time zero, trophozoites” and served as reference RNA for the time-course experiments. Of the remaining parasites, half the 30 ml flasks were treated with 10 nM WR99210 (1∶1,000 dilution of 10 µM WR99210 stock in 100% DMSO) and the other half were treated with solvent (final concentration of 0.1% DMSO). At various time-points (3 h to 24 h), infected erythrocytes were centrifuged down, and parasites were released by saponin treatment [75]. After two washes in PBS, the parasites were resuspended in lysis buffer (RNAqueouse Kit, Ambion) and RNA was isolated according to the manufacturer's instructions. As discussed above, 2 preparations of independently derived Dd2 were treated with solvent or with 10 nM WR99210. Similarly, 2 preparations of independently propagated B1G9 were treated with DMSO solvent or with 10 nM WR99210.

In Thailand, for each treatment, three 30 ml culture plates (90×15 mm) of TM4/8.2 parasites were set up at 4% hematocrit and 7–10% parasitemia. After two rounds of synchronization, an early trophozoites culture (approximately at 18–20 h post invasion) was treated with 0.5 µM pyrimethamine. The final concentration of DMSO solvent in each treatment was 0.1%. A culture containing 0.1% DMSO lacking drug was used as a control. After 2 h, 4 h, 8 h and 24 h of drug exposure, parasites were collected and extracted from erythrocytes by saponin lysis. Total RNA was purified from parasite cells using Trizol reagent (Invitrogen) according to the manufacturer's instructions. Experimental treatments were carried out from at least two independent cultures to wash out stochastic biological variation.

For RT-PCR based confirmation experiments, appropriate clones of independently seeded parasites were grown in 10 ml cultures and RNA was collected by RNAqueouse Kit (Ambion) according to the manufacturer's instructions. Contaminating DNA was removed from the total RNA samples using RQ1 RNase-Free DNase (Promega).

### Fluorescent cDNA preparation and hybridization

For each hybridization, 10 µg of total RNA was annealed with 5 µg pd(N)6 random primers (Amersham Biosciences Corp.) and reverse transcribed to produce aminoallyl-dUTP (Sigma)-labelled cDNA using StrataScript reverse transcriptase (Stratagene). Oligo-(dT)_21_ primer was used instead of the random hexamer for pyrimethamine treated samples from Thailand. The labelled cDNAs were coupled with either monoreactive-Cy3 or -Cy5 (Amersham Biosciences) as previously described [32]. Purified Cy3- and Cy5-labelled cDNAs were resuspended and mixed in 24 µl of hybridization solution containing 3× SSC, 0.2% SDS, 0.025 M HEPES and 0.75 µg/µl of poly A (Sigma) and hybridized on a *P. falciparum* 70-mer microarray for 16 h at 63°C.

### Data collection and analysis

After washing, slides were scanned in an Axon GenePix 4000B microarray scanner and the intensity of spots was quantified using GenePix Pro 3.0 Software (Axon Instruments, Inc.) as previously described [32]. Briefly, during the gridding process, images were inspected and visually problematic spots were manually flagged and removed. Spots with foreground intensity less than 2.1-fold of background intensity were considered too weak to be reliable and also removed. A scaled print-tip intensity-dependent lowess within-slide normalization was performed on each slide, followed by an across-slides normalization, using Aroma package version 0.89 [76] run in R project environment (http://cran.r-project.org). Differential expression and statistical analysis of Seattle data were done using a linear model method package Limma [77]. The criteria of the *p*-value of <0.05 and expression ratio≥2-fold change were employed for selection of differentially expressed genes. For Thailand data, statistically significant differences in gene expression were monitored using the Significance Analysis of Microarrays (SAM) program [78] and genes showing false discovery rate (FDR) = 0% and expression ratio of ≥1.5-fold in both directions were considered differentially expressed. Cluster analysis was performed using CLUSTER and visualized using TREEVIEW [79].

### Quantitative RT-PCR confirmation

Six of AFDG genes whose ratio of SD/SS was greater than 2 at 24 h of WR99210 exposure were selected for qRT-PCR validation. These were PFD1120c, PF10_0330, PFC0710w, PFB0915w, PFB0635w and MAL6P1.231. Three hundred nanograms of total RNA was primed with Oligo(dT)/random nanomers mix (at final concentration of 100 ng each/reaction) and converted to cDNA in 20 µl reactions using AffinityScript™ QPCR cDNA synthesis kit (Stratagene) as recommended by the manufacturer. The reverse transcription reaction was then diluted with 40 µl of nuclease-free PCR-grade water before using in the PCR amplification step. Real-time quantitative PCR was performed on a thermal cycler (DNA Engine, BioRad) equipped with a detector (Chromo4, BioRad). Primers were designed using PRIMER3 (http://www-genome.wi.mit.edu/genome_software/other/primer3.html) and optimized for annealing/extension temperature, concentration, and single product amplification. The designed primers are listed in Table S2. Amplifications were performed in 25 µl final volume, using 12.5 µl of 2× Brilliant® SYBR® Green QPCR Master Mix (Stratagene), 2 µl of the cDNA template, and 250 nM of each primer. Cycling conditions were: 10 min at 95°C and 40 cycles of 95°C for 15 sec followed by 58°C for 60 sec. The specificity of the amplifications was monitored by melting curve analysis and gel electrophoresis. The threshold cycle of fluorescence (Ct) was determined by Opticon Monitor 3 software (BioRad). The quantity of cDNA for each gene was normalized to the Seryl-tRNA synthetase (PF07_0073) concentration in each sample (ΔCt, Ct_gene_−Ct_PF07_0073_). Relative gene expression was calculated by 2^−ΔΔCt^ method [80], where ΔΔCt is the ratio of expression of each treatment relative to that of the trophozoite stage reference. Each PCR experiment was performed in duplicates with at least three RNA templates prepared from independent parasite cultures.

Analysis of the DHFR-TS oligos was performed with slight modifications to the procedure described above. Two hundred seventy nanograms of total RNA was used per cDNA synthesis reaction and then diluted with 25 µL of nuclease-free PCR-grade water. The annealing/extension temperature was reduced to 56°C. Analysis was performed using the Pfaffl method [81] with each primer pair's efficiency taken into account. Each experiment was performed in triplicate and the 24 h time-point was analyzed in three independent cultures. A representative run is shown and plotted error bars are the SEM of that run.

## Supporting Information

Figure S1 Structures of antifolates used to study changes in transcript levels in malaria parasites. (A) WR99210 used by the Seattle lab against sensitive clone Dd2 and resistant clone B1G9 carrying human DHFR. (B) Pyrimethamine used by the Bangkok lab against the sensitive clone TM4/8.2.
(0.24 MB TIF)

Figure S2 (A) Representative *M-A* plot of raw microarray data from SD24_rep1. In the analysis, data was derived from microarrays in which RNA from WR99210-treated (Cy5) parasite and RNA from reference pool (Cy3) control were compared. Raw data in the form of relative fluorescence intensity were log transformed and used to calculate *M* (difference in log intensities) and *A* (average log intensity) for each spot on the microarray. Most spots cluster around the zero line. Red and green spots indicate an *M* value of higher and lower than 1, respectively. (B) *M-A* plot of scaled print-tip and across slide normalized data. (C) Box-plot before normalization. (D) Box-plot of scaled print-tip and across slide normalized data.
(0.93 MB TIF)

Figure S3 Log_2_ (WR99210-treated/reference pool) comparison between replicates of representative experiment (SD24). (A) Log_2_ expression ratio comparison between SD24_rep1 and SD24_rep3 (technical replicate). SD24_rep3 is a dye swap experiment of SD24_rep1. (B) Log_2_ expression ratio comparison between SD24_rep1 and SD24_rep2 (biological replicate). (C) Log_2_ expression ratio comparison between SD24_rep1 and SD24_rep4 (biological replicate). SD24_rep4 is a dye swap experiment of SD24_rep2. Correlation between replicates demonstrates the high degree of reproducibility of the data, but biological replicates show greater variability in part due to stochastic changes in surface gene expression.
(0.46 MB TIF)

Figure S4 Genes whose expression changed permanently in resistant clone B1G9 comparing to sensitive clone Dd2 (with or without drug pressure). Each probe was tested for changes in expression in 4 comparisons (SS vs RS, SD vs RS, SS vs RD, and SD vs RD) at each time point. Probes were selected against these comparisons jointly, with multiple testing adjusted *p*<0.01.
(0.47 MB TIF)

Table S1 (0.01 MB PDF)

Table S2 (0.08 MB PDF)

Table S3 (0.03 MB XLS)

Table S4 (0.07 MB XLS)

Table S5 (0.05 MB XLS)

Table S6 (0.05 MB XLS)

Table S7 (0.05 MB XLS)
