# Supplementary figures and images for: A Genetically Hard-Wired Metabolic Transcriptome in Plasmodium falciparum Fails to Mount Protective Responses to Lethal Antifolates
Source: PLoS Pathog. 2008 Nov 21;4(11):e1000214. doi: 10.1371/journal.ppat.1000214 (PMC2581438; doi:10.1371/journal.ppat.1000214)

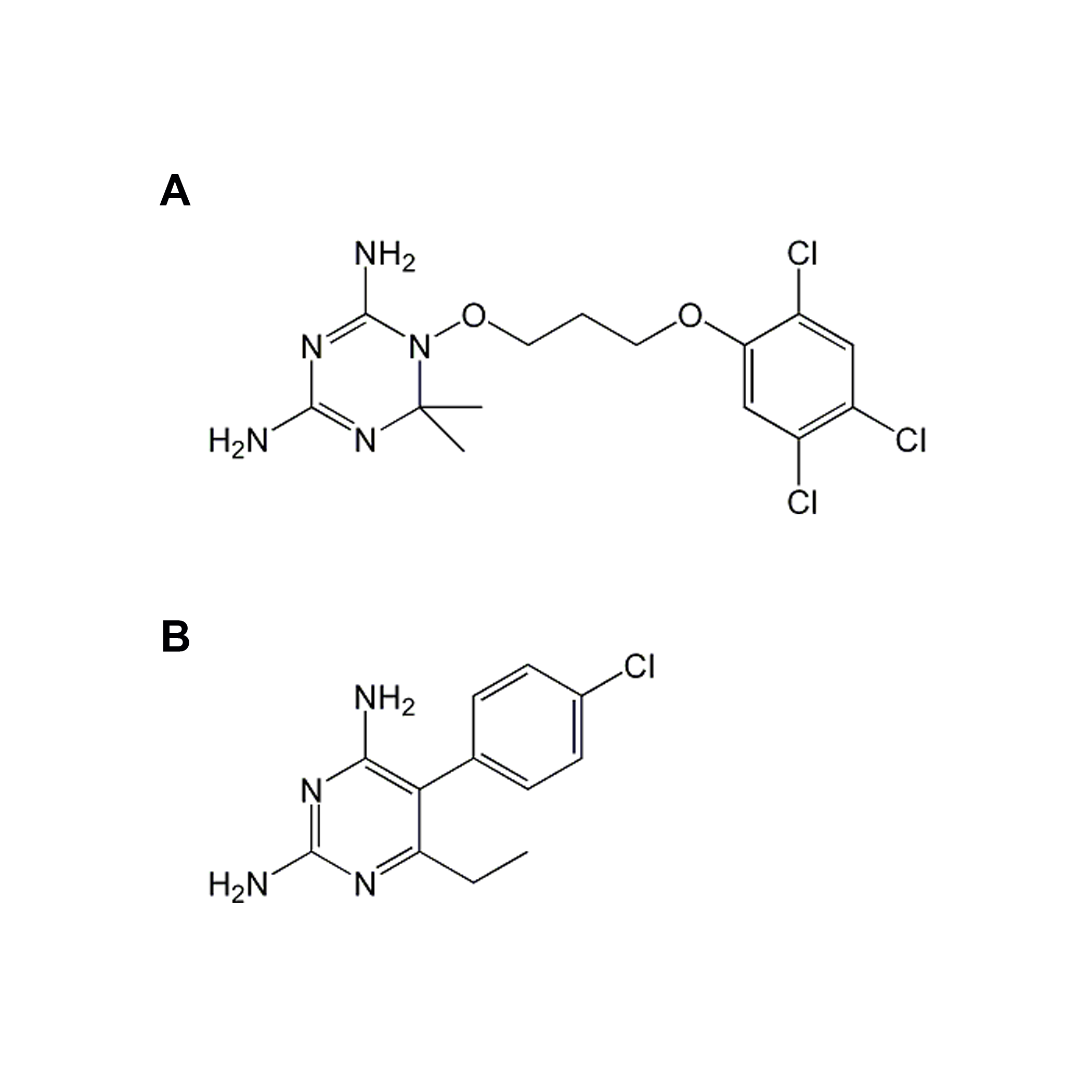

Supplement: Figure S1 — Structures of antifolates used to study changes in transcript levels in malaria parasites. (A) WR99210 used by the Seattle lab against sensitive clone Dd2 and resistant clone B1G9 carrying human DHFR. (B) Pyrimethamine used by the Bangkok lab against the sensitive clone TM4/8.2. (0.24 MB TIF) [file ppat.1000214.s001.tif]

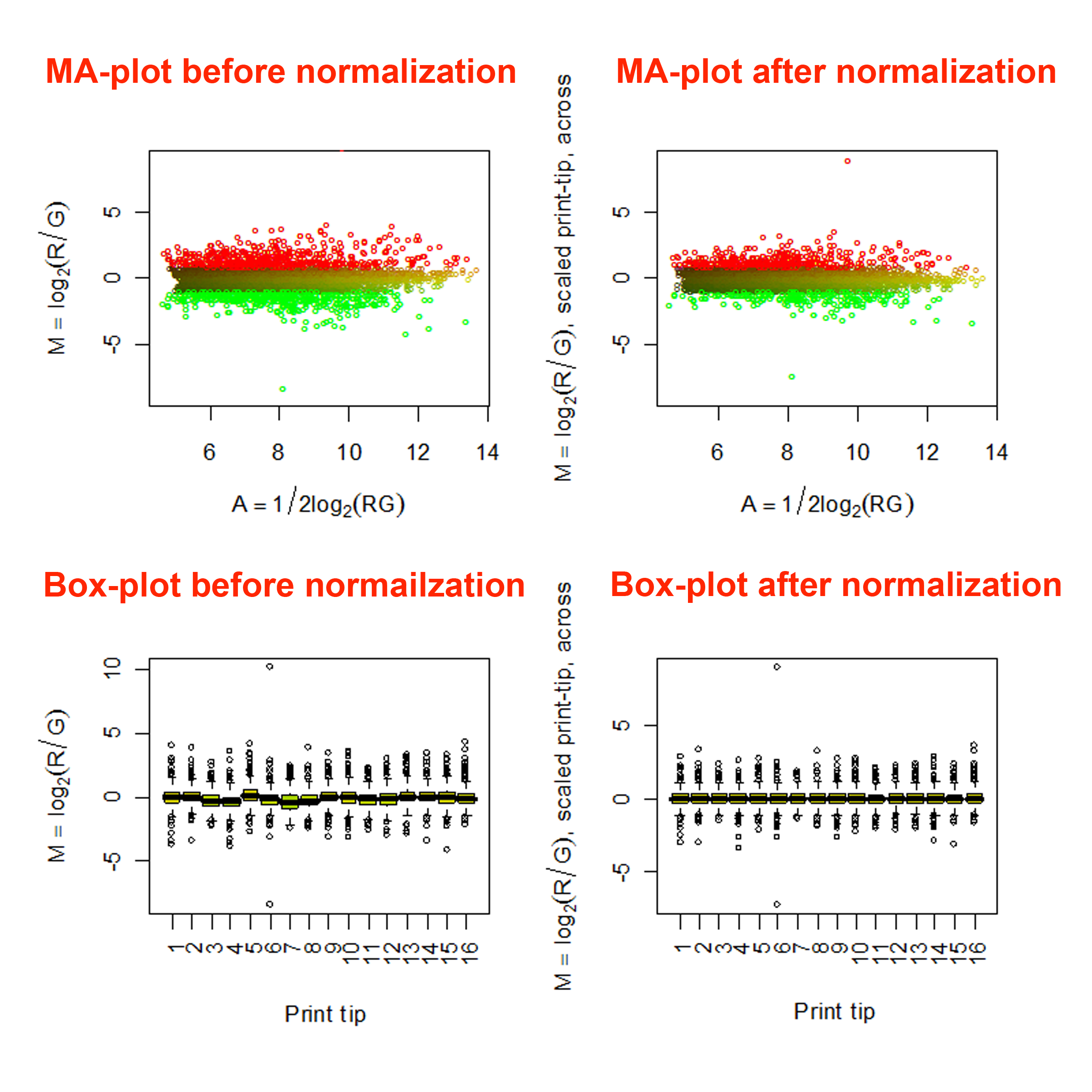

Supplement: Figure S2 — (A) Representative M-A plot of raw microarray data from SD24_rep1. In the analysis, data was derived from microarrays in which RNA from WR99210-treated (Cy5) parasite and RNA from reference pool (Cy3) control were compared. Raw data in the form of relative fluorescence intensity were log transformed and used to calculate M (difference in log intensities) and A (average log intensity) for each spot on the microarray. Most spots cluster around the zero line. Red and green spots indicate an M value of higher and lower than 1, respectively. (B) M-A plot of scaled print-tip and across slide normalized data. (C) Box-plot before normalization. (D) Box-plot of scaled print-tip and across slide normalized data. (0.93 MB TIF) [file ppat.1000214.s002.tif]

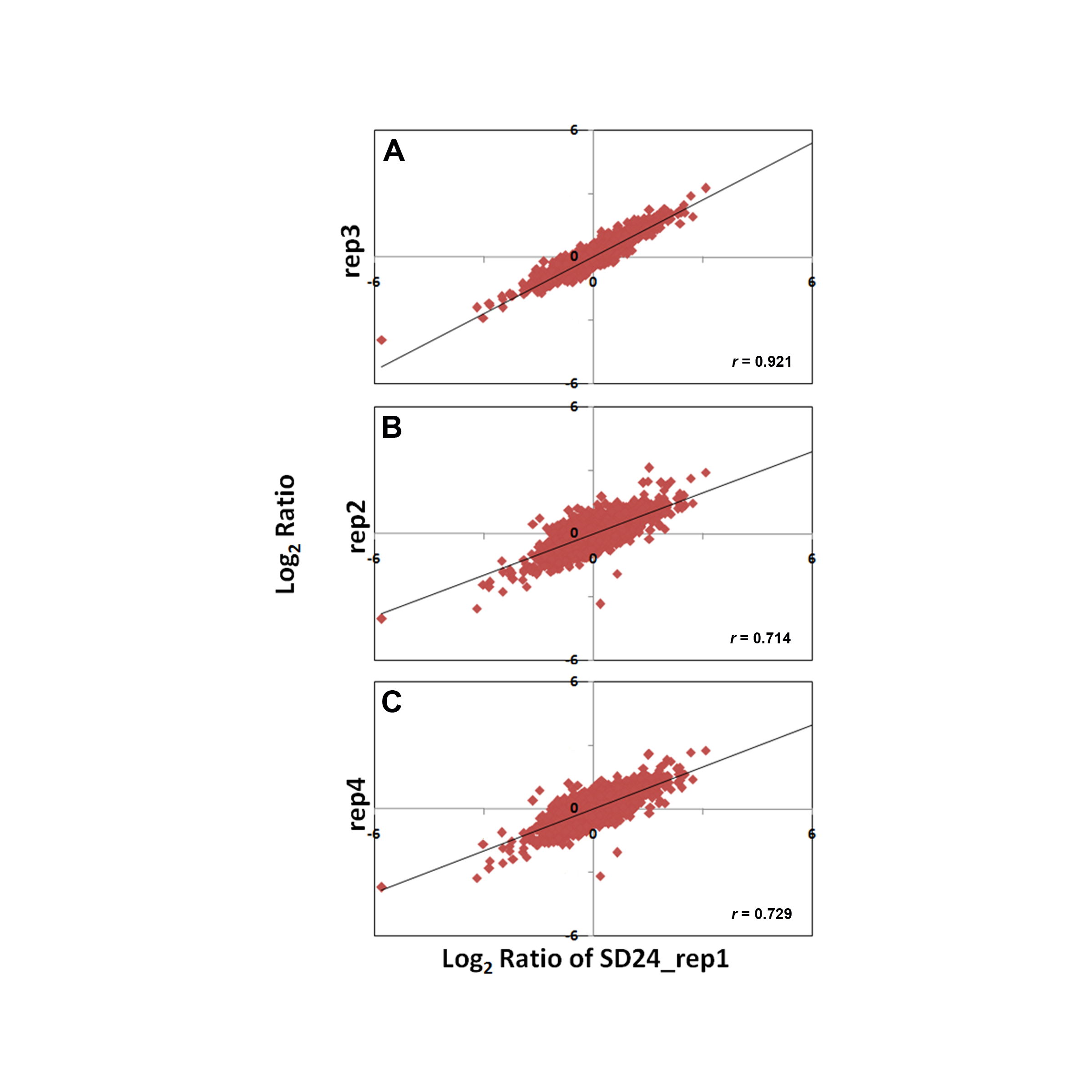

Supplement: Figure S3 — Log2 (WR99210-treated/reference pool) comparison between replicates of representative experiment (SD24). (A) Log2 expression ratio comparison between SD24_rep1 and SD24_rep3 (technical replicate). SD24_rep3 is a dye swap experiment of SD24_rep1. (B) Log2 expression ratio comparison between SD24_rep1 and SD24_rep2 (biological replicate). (C) Log2 expression ratio comparison between SD24_rep1 and SD24_rep4 (biological replicate). SD24_rep4 is a dye swap experiment of SD24_rep2. Correlation between replicates demonstrates the high degree of reproducibility of the data, but biological replicates show greater variability in part due to stochastic changes in surface gene expression. (0.46 MB TIF) [file ppat.1000214.s003.tif]

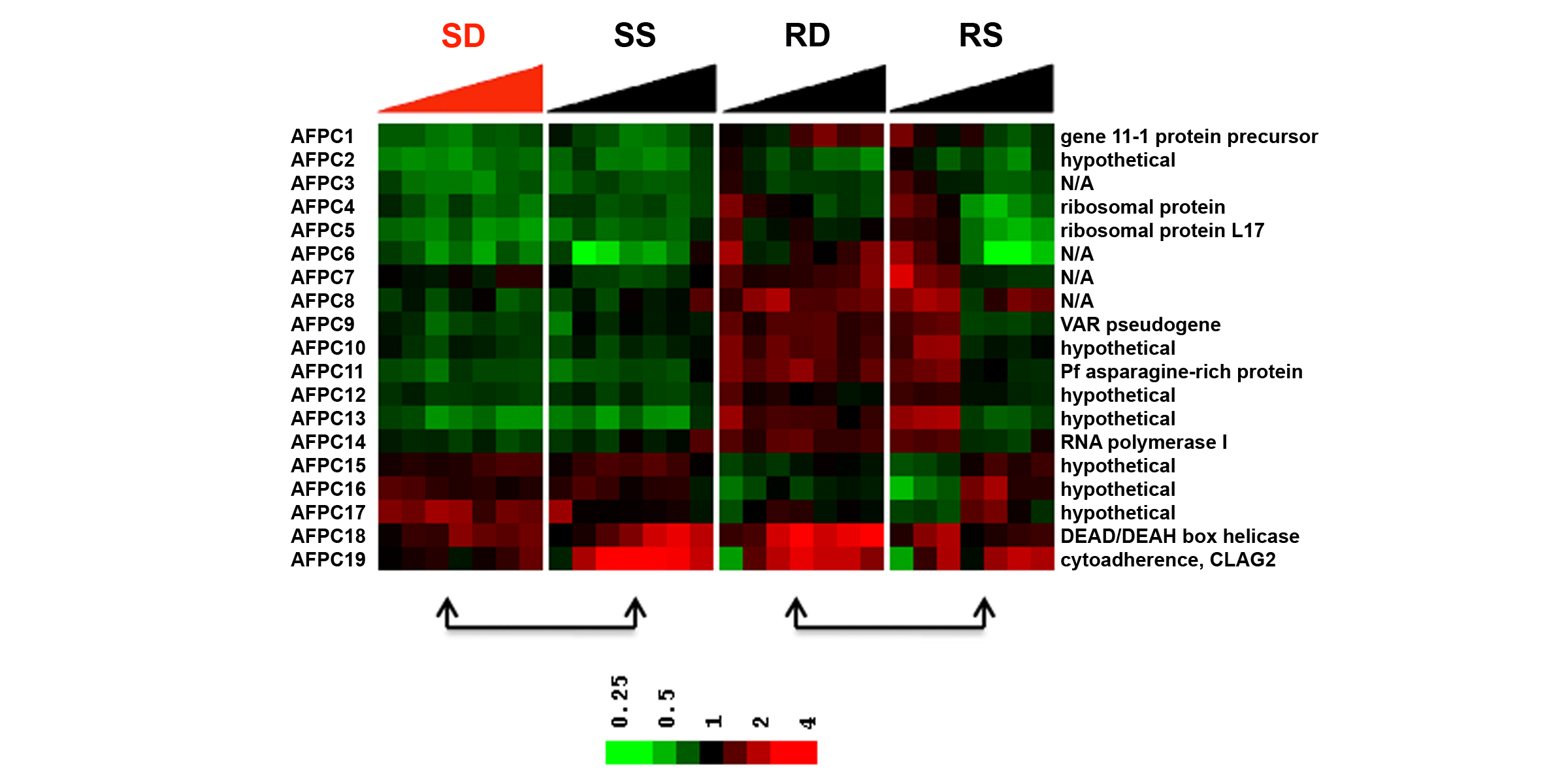

Supplement: Figure S4 — Genes whose expression changed permanently in resistant clone B1G9 comparing to sensitive clone Dd2 (with or without drug pressure). Each probe was tested for changes in expression in 4 comparisons (SS vs RS, SD vs RS, SS vs RD, and SD vs RD) at each time point. Probes were selected against these comparisons jointly, with multiple testing adjusted p<0.01. (0.47 MB TIF) [file ppat.1000214.s004.tif]
