# Supplementary material for: A Genetically Hard-Wired Metabolic Transcriptome in Plasmodium falciparum Fails to Mount Protective Responses to Lethal Antifolates
Source: PLoS Pathog. 2008 Nov 21;4(11):e1000214. doi: 10.1371/journal.ppat.1000214 (PMC2581438; doi:10.1371/journal.ppat.1000214)
Supplement: Table S1 — (0.01 MB PDF) [file ppat.1000214.s005.pdf]

Supplement Table S1: Oligos for DHFR-TS microarrays

**Oligo name**	**Sequence (5' to 3')**
D33539_76_13	TCTGCGACGTTTTCGATATTTATGCCATATGTGCATGTTGTAAGGTTGAAAGCAAAAATGAGGGGAAAAA
D33539_76_135	TTACCATGGAAATGTAATTCCCTAGATATGAAATATTTTTGTGCAGTTACAACATATGTGAATGAATCAA
D33539_76_264	GTAAATGATATGCCTAATTCTAAAAAATTACAAAATGTTGTAGTTATGGGAAGAACAAGCTGGGAAAGCA
D33539_76_411	GAAGATGTTTATATCATTAACAAAGTTGAAGATCTAATAGTTTTACTTGGGAAATTAAATTACTATAAAT
D33539_76_625	CTGTTAGCGATGTATATACTAGTAACAATACAACATTGGATTTTATCATTTATAAGAAAACGAATAATAA
D33539_76_764	CAAAGATACATGTCATATGAAAAAATTAACAGAATTTTACAAAAATGTAGACAAATATAAAATTAATTAT
D33539_76_1169	AACAAATGGTAATACGTTGTTAAATAAGAATGTAAGGATATGGGAAGCTAATGGTACTAGGGAATTTTTA
D33539_76_1260	AGAGAAGTTAACGATTTAGGACCTATTTATGGTTTTCAATGGAGACATTTCGGTGCTGAATATACAAATA
oPFD66954	GAAGAATTCTTTTGTGTGCATGGAATGTAAAAGATCTTGACCAAATGGCATTACCTCCTTGTCATATTTT
D33539_76	AGGGCTAGGAGTACCTTTTAATATTGCTTCTTATTCTATTTTTACTCATATGATTGCACAAGTCTGTAAT
D33539_76_1647	GCACATGTTTATAATAATCACATTGATAGTTTAAAAATTCAACTTAACAGAATACCCTATCCATTCCCAA
D33539_76_1756	TTACAATTTCGGATTTTACAATACAAAATTATGTTCATCATGAAAAAATTTCAATGGATATGGCTGCTTA
