# Supplementary material for: A Genetically Hard-Wired Metabolic Transcriptome in Plasmodium falciparum Fails to Mount Protective Responses to Lethal Antifolates
Source: PLoS Pathog. 2008 Nov 21;4(11):e1000214. doi: 10.1371/journal.ppat.1000214 (PMC2581438; doi:10.1371/journal.ppat.1000214)
Supplement: Table S2 — (0.08 MB PDF) [file ppat.1000214.s006.pdf]

Supplement Table S2: List of primers used for quantitative RT-PCR.

Gene	Primer name	Sequence (5′ to 3′)	Oligo mers	Product size (bp)
PFD1120c	*AFDG-U13*-F	GTCATGAGTAAAATTGACGAGATG	24	165
	AFDG-U13-R	ATCTTTTTCGTCACCTGATACTTC	24	
PF10_0330	*AFDG-U15*-F	ATCAGGATCTGTATGTCTTGACG	23	148
	AFDG-U15-R	CATTAGTAAGGAAGCAGCATCAC	23	
PFC0710w	*AFDG-U28*-F	GAGTGGTTCAGATCATATAAAATGG	25	141
	AFDG-U28-R	TTTCTTTACGTCTTCTCGAAACTC	24	
PFB0915w	*AFDG-D9*-F	AGTATTTGCTGCACCATTCATATC	24	174
	AFDG-D9-R	ACAAATGAATAAAACGGTCTCTCC	24	
PFB0635w	*AFDG-D11*-F	TCTGAATTCTTAATGGGTATGGTC	24	130
	AFDG-D11-R	AAACAGTACCTTTTGTTTCGGTAG	24	
MAL6P1.231	*AFDG-D21*-F	ATGTCATTAAGCCCATTTGATG	22	120
	AFDG-D21-R	CCTTGGTACTTTGAAATCTGCTAT	24	
PFD0830w	*D33539_76_13*-F	CTGCGACGTTTTCGATATTTATG	23	118
	D33539_76_13-R	TCCTTTATTTCCTAGACCTCTAAATGTG	28	
PFD0830w	*D33539_76_135*-F	ACACATTTAGAGGTCTAGGAAATAAAGG	28	181
	D33539_76_135-R	GAATTAGGCATATCATTTACATTATCCAC	29	
PFD0830w	*D33539_76_264*-F	AATGTAAATGATATGCCTAATTCTAAAAA	29	133
	D33539_76_264-R	TTAAGGTTCTAGACAATATAACATTTATCC	30	
PFD0830w	*D33539_76_411*-F	AAAAATTTAAACCTTTAAGCAATAGG	26	188
	D33539_76_411-R	CTTTTCTAAAAATTCTTGATAAACAACG	28	
PFD0830w	*D33539_76_625*-F	TTTATTATAGGAGGTTCCGTTGTTTATC	28	204
	D33539_76_625-R	CGTTTTCTTATAAATGATAAAATCCAATG	29	

Cont.

Gene	Primer name	Sequence (5′ to 3′)	Oligo mers	Product size (bp)
PFD0830w	*D33539_76_764*-F	GCTTGAAATATAAATATCATCCTGAA	26	117
	D33539_76_764-R	ATCCGAATTTACTTAAAACACCTACT	26	
PFD0830w	*D33539_76_1169*-F	AAGTCAATATTTCCCATTATTAACTACG	28	155
	D33539_76_1169-R	CTAAAAATTCCCTAGTACCATTAGCTTC	28	
PFD0830w	*D33539_76_1260*-F	AAGTTAACGATTTAGGACCTATTTATGG	28	108
	D33539_76_1260-R	TTTTTAATTGATCCACTCCTTTATTTTC	28	
PFD0830w	*oPFD66954*-F	TTGTGTGCATGGAATGTAAAAGAT	24	105
	oPFD66954-R	ATACATAATACATGATAATTTCCCATCG	28	
PFD0830w	*D33539_76*-F	TATGTATCAAAGATCATGTGATTTAGG	27	130
	D33539_76-R	ATTTCCTAAAACGTGTATGAACTGC	25	
PFD0830w	*D33539_76_1647*-F	CGCAGTTCATACACGTTTTAGG	22	122
	D33539_76_1647-R	TTTAATATCTGGATTTAATTTAAGTGTTGG	30	
PFD0830w	*D33539_76_1756*-F	TTGAAGATTTTACAATTTCGGATTTTAC	28	130
	D33539_76_1756-R	GTTCAGGTAATTTTGTCATCATTTGTTC	28	
PF07_0073	*stRNAs*-F	GGCACATGGAAAGGATATTAGAG	23	200
	stRNAs-R	TAGAAGCTGCGTTGTTTAAAGCTC	24	
